# Radiomics of pericoronary adipose tissue on computed tomography angiography predicts coronary heart disease in patients with type 2 diabetes mellitus

**DOI:** 10.1186/s12872-024-03970-4

**Published:** 2024-06-12

**Authors:** Shumei Miao, Feihong Yu, Rongrong Sheng, Xiaoliang Zhang, Yong Li, Yaolei Qi, Shan Lu, Pei Ji, Jiyue Fan, Xin Zhang, Tingyu Xu, Zhongmin Wang, Yun Liu, Guanyu Yang

**Affiliations:** 1 School of Computer Science and Engineering, Southeast University, Sipailou 2, Nanjing, 210096 Jiangsu China; 2 Department of Information, The First Affiliated Hospital of Nanjing Medical University, Nanjing, Jiangsu China; 3 Department of Medical Informatics, School of Biomedical Engineering and Informatics, Nanjing Medical University, Nanjing, Jiangsu China; 4 Department of Ultrasonic Department, The First Affiliated Hospital of Nanjing Medical University, Nanjing, Jiangsu China; 5 Department of Cardiovascular Medicine Department, The First Affiliated Hospital of Nanjing Medical University, Nanjing, Jiangsu China; 6 Department of Nutritional Department, The First Affiliated Hospital of Nanjing Medical University, Nanjing, Jiangsu China; 7 Department of Geriatrics endocrinology, The First Affiliated Hospital of Nanjing Medical University, Guangzhou Rd 300, Nanjing, 210096 Jiangsu China

**Keywords:** T2DM, CHD, CCTA, PCAT, Radiomics, Prediction

## Abstract

**Background:**

Diabetes is a common chronic metabolic disease. The progression of the disease promotes vascular inflammation and the formation of atherosclerosis, leading to cardiovascular disease. The coronary artery perivascular adipose tissue attenuation index based on CCTA is a new noninvasive imaging biomarker that reflects the spatial changes in perivascular adipose tissue attenuation in CCTA images and the inflammation around the coronary arteries. In this study, a radiomics approach is proposed to extract a large number of image features from CCTA in a high-throughput manner and combined with clinical diagnostic data to explore the predictive ability of vascular perivascular adipose imaging data based on CCTA for coronary heart disease in diabetic patients.

**Methods:**

R language was used for statistical analysis to screen the variables with significant differences. A presegmentation model was used for CCTA vessel segmentation, and the pericoronary adipose region was screened out. PyRadiomics was used to calculate the radiomics features of pericoronary adipose tissue, and SVM, DT and RF were used to model and analyze the clinical data and radiomics data. Model performance was evaluated using indicators such as PPV, FPR, AAC, and ROC.

**Results:**

The results indicate that there are significant differences in age, blood pressure, and some biochemical indicators between diabetes patients with and without coronary heart disease. Among 1037 calculated radiomic parameters, 18.3% showed significant differences in imaging omics features. Three modeling methods were used to analyze different combinations of clinical information, internal vascular radiomics information and pericoronary vascular fat radiomics information. The results showed that the dataset of full data had the highest ACC values under different machine learning models. The support vector machine method showed the best specificity, sensitivity, and accuracy for this dataset.

**Conclusions:**

In this study, the clinical data and pericoronary radiomics data of CCTA were fused to predict the occurrence of coronary heart disease in diabetic patients. This provides information for the early detection of coronary heart disease in patients with diabetes and allows for timely intervention and treatment.

## Background

Diabetes is a common chronic metabolic disease, and its incidence is second only to cardiovascular disease and malignant tumors. As diabetes progresses, it can cause damage to the vascular endothelium and promote the formation of atherosclerosis, which leads to cardiovascular disease. Coronary heart disease (CHD) is a serious complication of diabetes mellitus with high disability and fatality rates [1–3]. Coronary angiography (CAG) examination is the gold standard for the diagnosis of CHD. Due to its invasive nature and certain complications, CAG is generally used as a routine screening method before the treatment of coronary artery disease. Coronary computer tomography angiography (CCTA), as a noninvasive imaging mode, has been widely used in the evaluation and diagnosis of patients with suspected CHD [4, 5]. CCTA can not only reliably evaluate luminal stenosis and its functional significance but also accurately evaluate the morphology and composition of plaques and identify vulnerable plaques, which has extremely important significance for guiding the clinical management of patients with CHD. Pericoronary fat based on CCTA is closely related to coronary plaque and stenosis. The pericoronary fat attenuation index is a novel noninvasive imaging biomarker that reflects the spatial changes in pericoronary adipose tissue (PCAT) attenuation and pericoronary inflammation in CCTA images [6, 7].

Radiomics is a high-throughput extraction of a large number of image features from radiographic images and a deeper mining, prediction and analysis of common images [8, 9]. Radiomics was originally mainly applied in oncology research. In recent years, relevant studies have revealed that it has certain potential application value in the diagnosis of CHD, prediction of adverse cardiovascular events, monitoring of disease progression and evaluation of prognosis [10–12]. At present, there have been related studies on clinical data and medical image analysis of diabetes mellitus and coronary heart disease [13–15]. However, few studies have focused on the association between PCAT around coronary plaques and the possibility of CHD events in type 2 diabetes mellitus (T2DM) patients. Therefore, the purpose of this study was to investigate the ability of vascular and pericoronary fat radiomics results based on CCTA to predict the occurrence of CHD in diabetic patients.

## Methods

In this section, we mainly describe the clinical dataset used in the experiment and all the details of our proposed method.

### Dataset

**Fig. 1:**
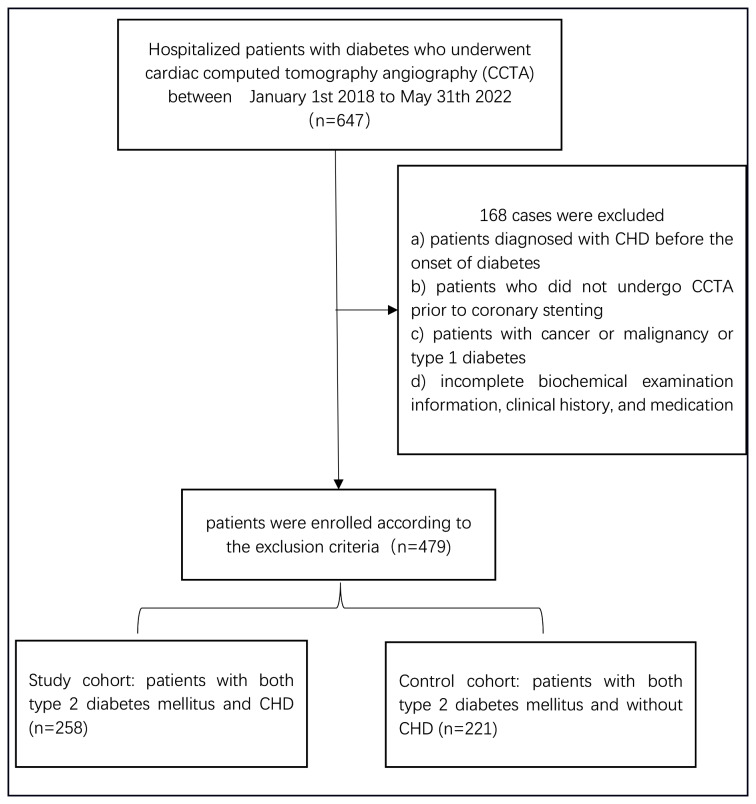
The patient enrollment flow diagram

Clinical data of patients were collected from the clinical data center and image center of the First Affiliated Hospital of Nanjing Medical University. The population of this study was first enrolled with hospitalized patients with diabetes who underwent cardiac computed tomography angiography (CCTA), and 647 hospitalized patients were retrieved from January 1st 2018 to May 31st 2022. Then, patients diagnosed with CHD before the onset of diabetes, patients who did not undergo CCTA prior to coronary stenting, patients with cancer or malignancy or type 1 diabetes, and patients with incomplete biochemical examination information, clinical history, and medication were excluded. A total of 258 + 221 patients were enrolled according to the exclusion criteria; 258 patients with both CHD and diabetes and 221 patients with diabetes but no CHD as controls were finally enrolled in this study. The flow diagram of this study is illustrated in Fig. 1. The study was approved by the Ethics Committee of the First Affiliated Hospital of Nanjing Medical University (2019-SR-153). As this study is a retrospective study and the contents of the study do not involve personal privacy, the Ethics Committee of the First Affiliated Hospital of Nanjing Medical University waived the requirement for written informed consent.

### B. Statistical methods

The statistical analysis was performed using R software version 4.1.2 (R Foundation for Statistical Computing, Vienna, Austria). Normality was checked by the Kolmogorov‒Smirnov test. Normally distributed variables are described as the mean±standard deviation, and nonnormally distributed variables are described as the median ± (25th-75th percentiles). The χ^2^ test was used to compare categorical variables. Student’s t test was used to compare continuous variables. Basic characteristics with *p* < 0.05 were considered statistically significant and included in the following modeling. Radiomics features with a p value < 0.001 were included for model establishment.

### C. Image acquisition and segmentation

The CCTA images of all patients in this study were obtained from Siemens and GE computerized tomography instruments of the First Affiliated Hospital of Nanjing Medical University. The original data were maintained in DICOM data format with 512*512 dimensions, the patients’ personal information was desensitized, and the detailed configuration information of DICOM message headers was retained for the subsequent extraction and analysis of data feature values. Deep learning algorithms have made long-term progress in the segmentation of various tissues and organs of medical images [16–19]. However, the existing methods have a large proportion of tissue structures and clear edges, while the coronary vessels to be segmented in this study have a small proportion, long and thin vessels with fuzzy edges, and the labeling of coronary vessels needs to be marked layer by layer, which is laborious and difficult to obtain a lot of labeling information. Therefore, we adopted the method of coronary artery segmentation proposed our research group yaolei et al. [20]. We used the easily obtained central axis as weak supervision information, proposed an examinee-examiner network, and used customized prior conditions to guide and constrain segmentation to deal with weak supervision with fewer intracranial labels to achieve accurate segmentation of coronary artery lumens and obtain the ROI of the coronary vessel area, to achieve accurate segmentation results. Based on the training model of the training dataset, the dataset used in this study was transferred. The automatic extraction of the coronary artery was realized.

#### D. Feature extraction

PCAT was defined as all voxels with CT attenuation between − 190 and − 30 Hounsfield units (HUs) located within a radial distance from the outer coronary wall equal to the diameter of the vessel [12, 21]. In this study, the feature extraction of images was carried out through the open-source software package PyRadiomics [22–24]. PyRadiomics is implemented by the Python algorithm, which is a popular open-source language for scientific computing. Python version 3.6.4 is adopted in this study. PyRadiomics is a standardized and open platform with a large number of standardized feature extraction algorithms, which is often used for the extraction and processing of imaging features of medical image data. In this study, seven standardized characteristic classes, including first-order statistics, shape features, gray-level cooccurrence matrix (GLCM), gray-level run length matrix (GLRLM), gray-level size zone matrix (GLSZM), gray-level dependence matrix (GLDM), and neighboring gray tone difference matrix (NGTDM), were mainly calculated. A total of 1,037 radiomic parameters were calculated for each PCAT segmentation. Figure 1 shows the process of CCTA image acquisition, segmentation, radiomics feature extraction of the pericoronary adipose region, feature screening and model construction for the studied patients.

### E. Model construction

#### Support vector machine (SVM)

SVM is a supervised machine learning algorithm based on statistical learning theory using the concept of structural risk minimization. It has been applied to many medical diagnoses and disease classifications. The computational time complexity of the SVM algorithm varies with different kernels, ranging from O (n2) to O (n3), where n represents the number of training sessions.

#### Decision tree (DT)

In the DT algorithm, instances (data points) are classified by sorting them based on feature values. In this study, the Gini index algorithm was used to identify the corresponding threshold to split the input data into subbranches. The computation time complexity of the DT algorithm is O(n*log(n)*f), where n represents the number of training capital and f represents the number of features.

#### Random forests (RF)

RT is an ensemble-type classification method that tends to perform better than traditional decision tree classification methods. The computational time complexity of the RF algorithm is O(n*log(n)fk), where n represents the number of training samples, f represents the number of features, and k represents the number of trees. We used 500 trees to predict two target classes, the occurrence or not occurrence of CHD in diabetic patients.

We developed SVM, DT and RF models to identify risk factors for CHD occurrence in type 2 diabetes patients. We used the package e1071 in the open-source software R 4.1.2 to perform SVM models, and the DT and RF were implanted using the packages rpart and randomForest, respectively. For the preprocessing of input data before modeling, since Stochastic resonance theory explains the use of noise constructively to enhance the input signal [25–27], we did not perform filtering operation. Instead, the input data is directly normalized and then modeled.

### F. Performance evaluation

Parameters such as sensitivity, specificity, positive predictive value (PPV), negative predictive value (NPV), false positive rate (FPR), false negative rate (FNR), F score and accuracy (AAC) were calculated. The receiver operating characteristic (ROC) curve area was calculated to observe the relationship between PCAT around coronary plaque and internal radiomics information based on CCTA and the possibility of CHD events in diabetic patients.

**Fig. 2:**
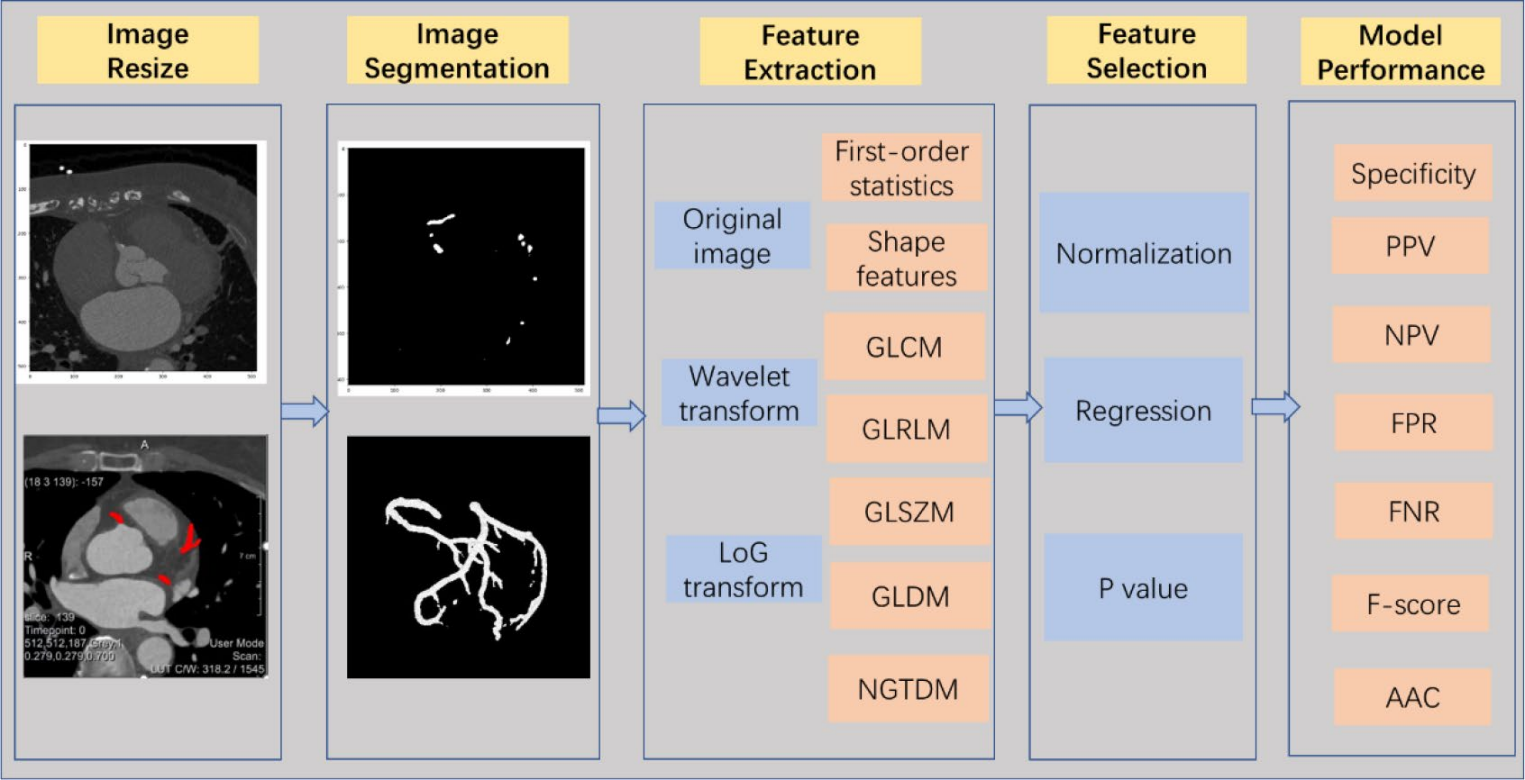
The flowchart of the current study

Figure 2 shows the full flowchart of the current study. First, the collected CCTA images are resampled and segmented with method in section C. Then, the proposed regions are used as templates for feature extraction using radiomics with method in section D. For the diameter range around the extracted blood vessels and the CT attenuation value is the range area describing the tissue, the PCAT was extracted using the PyRadiomics package. After that, feature selection is performed using statistical methods. The models are constructed using SVM, DT, and RF methods, and the performance of the models is evaluated.

## Results

### Clinical characteristics

Our patients’ mean age was 66.85 ± 11.42 years, and 65.76% of them were male. The clinical characteristics and morphological parameters of the patients in the two cohorts are illustrated in Table 1. Patients with both diabetes and CHD (study group) and patients with diabetes but no CHD (comparison group) were analyzed by common factors such as sex, age, hypertension, blood pressure, blood biochemical function test, height and weight. We observed that patients in the study group were older than those in the comparison group (71.35±10.18 vs. 61.43±10.52, *P* < 0.001). Patients with both diabetes and CHD had a lower diastolic pressure (74.36±11.50, 77.90±11.03, *p* < 0.001) and lower HDLC (1.04±0.265, 1.12±0.398, *p* = 0.012). In contrast, gender, smoking status, alcohol consumption, family history of coronary disease, and systolic pressure were not significantly different between these two cohorts.

**Table 1:** The general characteristics of the study population

	study group (*n* = 258)	comparison group (*n* = 221)	*p* value
**Sex**			0.304
Male, n (%)	175(67.83)	140(63.35)	
Female, n (%)	83(32.17)	81(36.65)	
**Age, yr**	71.35 ± 10.18	61.43 ± 10.52	**< 0.001** ^***^
**Hypertension**			**< 0.001** ^***^
No, n (%)	19(7.36)	165(74.66)	
Yes, n (%)	239(92.64)	56(25.34)	
**Smoke**			0.611
No, n (%)	173(67.05)	153(69.23)	
Yes, n (%)	85(32.95)	68(30.77)	
**Alcohol**			0.070
No, n (%)	189(73.26)	145(65.61)	
Yes, n (%)	69(26.74)	76(34.39)	
**Family history of Coronary**			0.089
No, n (%)	252(97.67)	220(99.55)	
Yes, n (%)	6(2.33)	1(0.45)	
**Family history of Diabetes**			**0.008** ^**^
No, n (%)	238(92.25)	187(84.62)	
Yes, n (%)	20(7.75)	34(15.38)	
**Drug for Hypoglycemic**			**< 0.001** ^***^
No, n (%)	129(50)	80(36.2)	
Yes, n (%)	129(50)	141(63.8)	
**Systolic pressure, mmHg**	134.67 ± 17.48	132.05 ± 17.00	0.098
**Diastolic pressure, mmHg**	74.36 ± 11.50	77.90 ± 11.03	**< 0.001** ^***^
**Height, cm**	166.12 ± 7.95	168.17 ± 7.73	**0.004** ^**^
**Weight, kg**	69.86 ± 34.98	72.49 ± 12.87	0.262
**GLU**	6.16 ± 2.169	6.21 ± 2.068	0.789
**HDLC**	1.04 ± 0.265	1.12 ± 0.398	**0.012** ^**^
**LDLC**	2.12 ± 0.750	2.64 ± 0.841	**< 0.001** ^***^
**TC**	3.60 ± 1.022	4.39 ± 1.463	**< 0.001** ^***^
**TG**	1.37 ± 0.837	1.70 ± 1.085	**< 0.001** ^***^
**HBA1C**	7.02 ± 1.306	7.02 ± 1.537	0.993

*T2DM: type 2 diabetes mellitus; CHD: coronary heart disease; GLU: glucose; HDLC: high density lipoprotein; LDLC: low density lipoprotein cholesterol; TC: total cholesterol; TG: triglyceride; HBA1C: glycosylated hemoglobin

### Radiomic feature selection

**Fig. 3:**
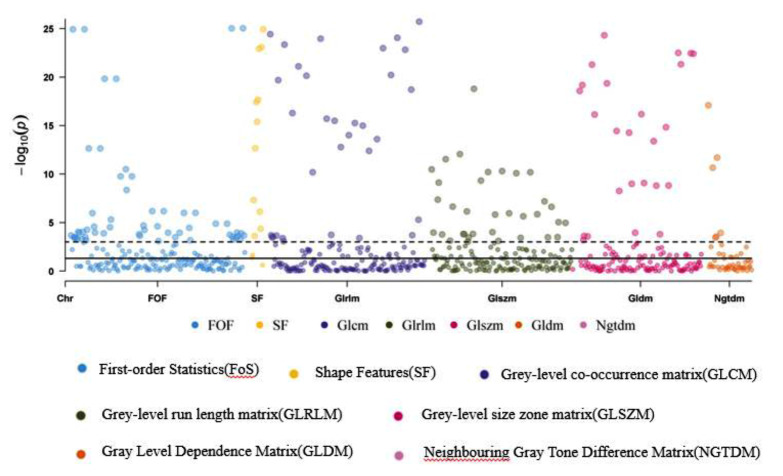
Negative logarithm of p values for comparison between diabetes with CHD and diabetes without CHD are plotted on the y axis, each of 1037 radiomic parameters are showed on the x axis. The broken line indicates the p value of 0.001, and 190 parameters above the line were considered statistically significant

Of all 1,037 calculated radiomic parameters, 18.3% (190 of 1037) showed a significant difference (*p* < 0.001) between the 2 groups. Specifically, 28.28% (56 of 198) of first-order parameters, 78.57 (11 of 14) of shape parameters, 7.95% (21 of 264) of GLCM parameters, 18.18 (32 of 176) of GLRLM parameters, 21.02 (37 of 176) of GLSZM parameters, 17.53 (27 of 154) of GLDM parameters, and 10.91 (6 of 55) of NGTDM parameters were significant (Fig. 3).

### Identification of diabetes patients with CHD using ML

Three different kinds of machine learning models, including support vector machine (SVM), decision tree (DT), and random forests (RF), were used to investigate the association of coronary heart disease occurrence in diabetic patients. We built a dataset with significantly different clinical features, including age, hypertension, family history of diabetes, diastolic pressure, height, HDLC, LDLC, TC and TG, from Table 1 as C-data. Different from this dataset, we set CCTA-based imaging biomarkers: significant radiomic features in the coronary vessel section as V-data. Then, we set the PCAT radiomics phenotype with a significant difference from the V-data as P-data. We used three combinations of clinical information, radiomics information of the vascular region, and radiomics information of perivascular fat to observe the different prediction effects of different datasets.

Table 2 shows the recognition effects of different datasets using different models. We can see that the datasets combining clinical information, vascular internal radiomics information and pericoronary vascular fat radiomics information have the highest ACC values under different machine learning models. The methods using support vector machines all performed best in specificity, sensitivity and accuracy in this dataset. Therefore, it also verified our view that the peri-coronary adipose tissue omics data reflect the perivascular inflammatory characteristics, which is helpful to improve CHD patients’ discrimination from diabetic patients.

Furthermore, the study tries to analyze the elements that have a large influence on the results during the modeling process. The paper takes the analysis process of random forest as an example. The top important variables in 3 kinds of datasets are shown in.

**Table 2:** The classifying results from different datasets combinations using three models

Types of algorithm	Data sets	Sensitivity (%)	Specificity (%)	PPV (%)	NPV (%)	FPR (%)	FNR (%)	F-Score (%)	ACC (%)	AUC	95% CI
Support vector machine	C_data	86.30%	73.24%	76.83%	83.87%	26.76%	13.70%	67.02%	79.86%	0.804	0.738–0.869
	C_data + V_data	94.05%	95.00%	96.34%	91.94%	5.00%	5.95%	71.82%	94.44%	0.941	0.902–0.981
	C_data + V_data + P_data	95.18%	95.08%	96.34%	93.55%	4.92%	4.82%	71.82%	95.14%	0.949	0.913–0.986
Decision tree	C_data	93.90%	72.58%	81.91%	90.00%	27.42%	6.10%	75.49%	84.72%	0.832	0.771–0.894
	C_data + V_data	91.46%	83.87%	88.24%	88.14%	16.13%	8.54%	71.77%	88.19%	0.885	0.831–0.938
	C_data + V_data + P_data	91.46%	85.48%	89.29%	88.33%	14.52%	8.54%	71.43%	88.89%	0.885	0.831–0.938
Random forest	C_data	84.09%	85.71%	90.24%	77.42%	14.29%	15.91%	70.48%	84.72%	0.838	0.777-0.900
	C_data + V_data	91.76%	93.22%	95.12%	88.71%	6.78%	8.24%	71.56%	92.36%	0.919	0.873–0.965
	C_data + V_data + P_data	90.80%	94.74%	96.34%	87.10%	5.26%	9.20%	71.82%	92.36%	0.917	0.870–0.964

* C_data as clinical data, V_data as vessel radiomics, P_data as pericoronary fat radiomics

**Fig. 4:**
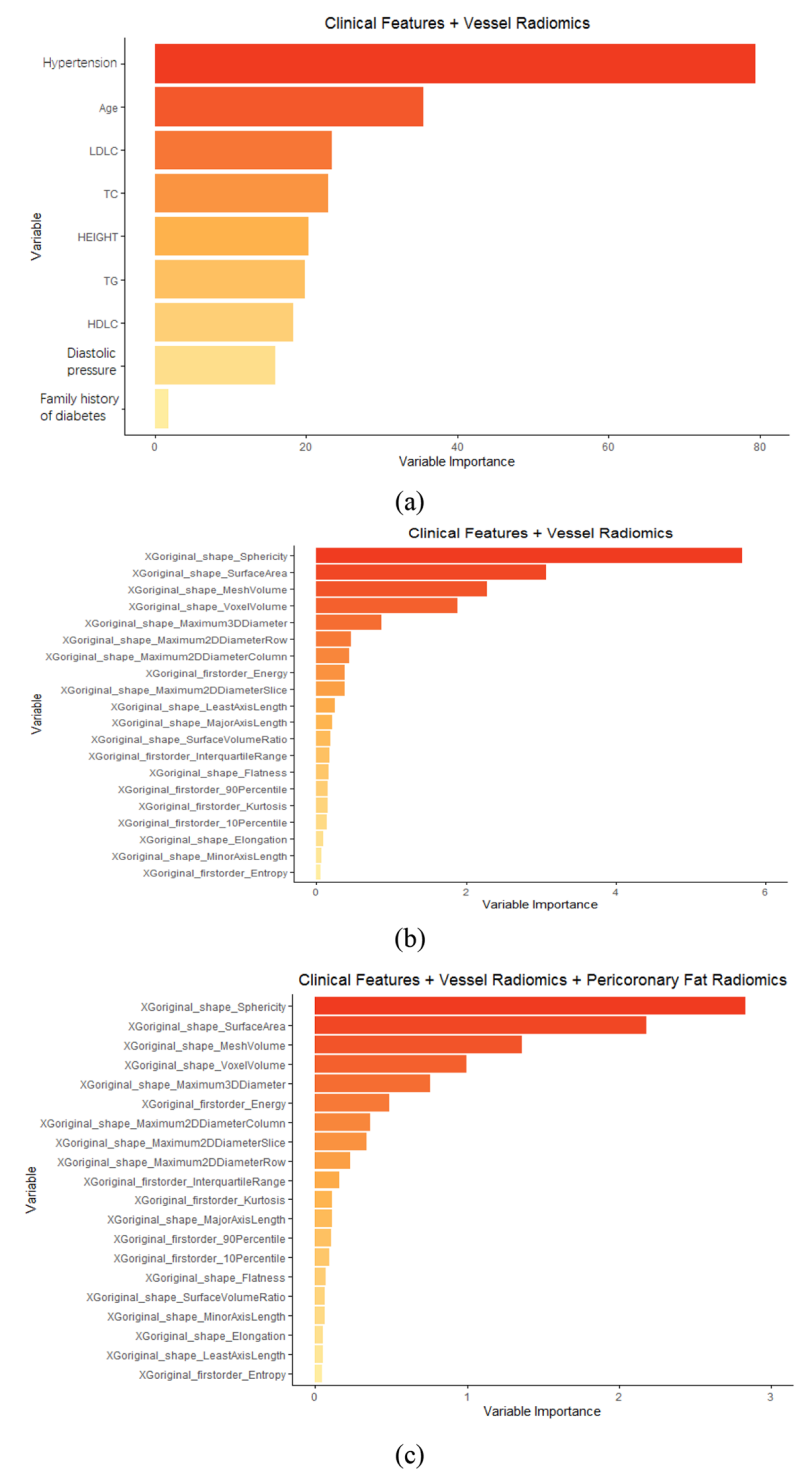
Ranking of variables in 3 kinds of data sets in random forest model. (1) clinical data alone; (2) clinical data and coronary vessel radiomics feature; (3) clinical data, coronary vessel radiomics feature and perivascular fat radiomics features

Figure 4A and C, and the importance for the classification of CHD from diabetic patients was ranked. As seen from the ranking, once the radiomics dataset is integrated with the clinical dataset, the radiomics features are more relevant, especially in terms of shape features and first-order features, which also reflects the high impact of CCTA-based image features on disease diagnosis.

## Discussion

In this study, the clinical data and pericoronary radiomics data of CCTA were fused to predict the occurrence of coronary heart disease in diabetic patients. This provides information for the early detection of coronary heart disease in patients with diabetes and allows for timely intervention and treatment. Coronary artery inflammation is an early manifestation of cardiovascular disease. Blood vessels in an inflammatory state release inflammatory factors that diffuse to pericoronary fat, resulting in local inhibition of fat formation and promotion of local fat decomposition through a series of processes. Antonopoulos et al. [6] suggested a “bidirectional” interaction between coronary artery wall inflammation and pericoronary adipose tissue (PCAT). The relationship between PCAT inflammation and the CT attenuation index was confirmed through biopsies. When there is inflammation in the blood vessels, the inflammatory lesions affect the CT value of PCAT on CCTA images through paracrine action. T2DM is a metabolic disorder in which abnormal insulin activity leads to disturbances in fat metabolism within the body. T2DM is strongly correlated with CHD, and one crucial mechanism is the chronic hyperglycemia-induced vascular inflammation that contributes to the development of atherosclerosis. Inflammation plays a pivotal role as a regulatory factor in metabolism and diabetes-related CHD. Research suggests that T2DM patients are twice as likely to develop CHD compared to nondiabetic individuals [28–30]. T2DM patients are prone to the formation of high-risk plaques when compared to those without T2DM, despite the presence of proactive treatment approaches and improved diagnostics. Consequently, the risk of cardiovascular complications remains significantly elevated in T2DM patients. Therefore, the early identification and prevention of CHD lesions hold significant importance for individuals with T2DM.

To the naked eye, the perivascular adipose tissue appears uniformly black, making it difficult to distinguish. However, through omics processing, colors can be assigned to the fat, revealing noticeable contrasts. Radiomics substantially enhances the quantity of quantitative data that can be extracted from CT images. It involves extracting thousands of imaging features that are imperceptible to the human eye from a specific region of interest. These features are then used to generate a vast dataset, which is subsequently analyzed to reveal imaging patterns linked to clinical traits or outcomes. Currently, PCAT has been demonstrated to be capable of predicting plaque progression in coronary arteries and distinguishing types of CHD [21, 31–33]. Radiomics can extract a multitude of image features from a given region of interest, far beyond parameters discernible by the human eye [18]. It has emerged as a novel imaging biomarker for atherosclerosis in coronary arteries. This study further analyzed the utilization of PCAT-based radiomic features as detection markers for coronary heart disease in diabetic patients. Through a comparison of three modeling approaches, the predictive efficacy of PCAT was validated. When combined with clinical and vascular information, its effectiveness was further substantiated. AI-assisted measurement of PCAT parameters is rapid, convenient, noninvasive, and easily scalable in clinical settings. This approach can provide early warnings for coronary heart disease in high-risk diabetic populations, ultimately improving their subsequent quality of life.

This study has the following limitations. First, it is a retrospective study with a single center and a small sample size. Second, although CAG is commonly used as the gold standard for diagnosing coronary heart disease (CHD) and as a common treatment method in clinical practice, a large number of diabetic patients do not undergo CAG before the occurrence of obvious CHD symptoms. In this study, the presence of CHD was determined based on comprehensive patient medical records, examinations, and diagnoses. In future studies, it is necessary to address the limitations of a single center and small sample size by expanding the study population. Additionally, enriching clinical information and ensuring accurate patient selection should be further emphasized.

## Conclusion

In this study, the clinical data and pericoronary radiomics data of CCTA were fused to predict the occurrence of coronary heart disease in patients with diabetes mellitus. The high-dimensional image features calculated by radiomics were used to extract the parameters that could not be identified by the naked eye. Three machine learning models were selected for classification and comparison. The well-performed classification results confirmed that the radiomics of pericoronary adipose tissue can reflect the perivascular inflammatory features and help to identify CAD patients among diabetic patients.

## Data Availability

The datasets used and/or analysed during the current study are available from the corresponding author on reasonable request.

## Abbreviations

CCTA: coronary computed tomography angiography
T2DM: type 2 diabetes mellitus
CHD: coronary heart disease
PCAT: pericoronary adipose tissue
CAG: Coronary angiography
