# Correction: Radiomics of pericoronary adipose tissue on computed tomography angiography predicts coronary heart disease in patients with type 2 diabetes mellitus

**DOI:** 10.1186/s12872-024-04037-0

**Published:** 2024-07-16

**Authors:** Shumei Miao, Feihong Yu, Rongrong Sheng, Xiaoliang Zhang, Yong Li, Yaolei Qi, Shan Lu, Pei Ji, Jiyue Fan, Xin Zhang, Tingyu Xu, Zhongmin Wang, Yun Liu, Guanyu Yang

**Affiliations:** 1 School of Computer Science and Engineering, Southeast University, Sipailou 2, Nanjing, 210096 Jiangsu China; 2 Department of Information, The First Affiliated Hospital of Nanjing Medical University, Nanjing, Jiangsu China; 3 Department of Medical Informatics, School of Biomedical Engineering and Informatics, Nanjing Medical University, Nanjing, Jiangsu China; 4 Department of Ultrasonic Department, The First Affiliated Hospital of Nanjing Medical University, Nanjing, Jiangsu China; 5 Department of Cardiovascular Medicine Department, The First Affiliated Hospital of Nanjing Medical University, Nanjing, Jiangsu China; 6 Department of Nutritional Department, The First Affiliated Hospital of Nanjing Medical University, Nanjing, Jiangsu China; 7 Department of Geriatrics endocrinology, The First Affiliated Hospital of Nanjing Medical University, Guangzhou Rd 300, Nanjing, 210096 Jiangsu China

**Correction: BMC Cardiovascular Disorders (2024) 24:300**

10.1186/s12872-024-03970-4

Following publication of the original article [1], the affiliation details for Corresponding Author Guanyu yang were incorrectly given as ‘^1^ School of Computer Science and Engineering, Southeast University, Sipailou 2, Nanjing 210096, Jiangsu, China; ^4^ Department of Ultrasonic Department, The First Affiliated Hospital of Nanjing Medical University, Nanjing, Jiangsu, China’ but should have been ‘^1^ School of Computer Science and Engineering, Southeast University, Sipailou 2, Nanjing 210096, Jiangsu, China’. 

The original article has been corrected.
